# Multi-scale complexity analysis of muscle coactivation during gait in children with cerebral palsy

**DOI:** 10.3389/fnhum.2015.00367

**Published:** 2015-07-22

**Authors:** Wen Tao, Xu Zhang, Xiang Chen, De Wu, Ping Zhou

**Affiliations:** ^1^Neuromuscular Control Laboratory, Department of Electronic Science and Technology, University of Science and Technology of ChinaHefei, China; ^2^Department of pediatrics, First Affiliated Hospital of Anhui Medical UniversityHefei, China; ^3^Department of Physical Medicine and Rehabilitation, University of Texas Health Science Center at Houston, TIRR Memorial Hermann Research CenterHouston, TX, USA

**Keywords:** cerebral palsy, electromyography, gait analysis, multi-scale analysis, multivariate sample entropy

## Abstract

The objective of this study is to characterize complexity of lower-extremity muscle coactivation and coordination during gait in children with cerebral palsy (CP), children with typical development (TD) and healthy adults, by applying recently developed multivariate multi-scale entropy (MMSE) analysis to surface electromyographic (EMG) signals. Eleven CP children (CP group), eight TD children and seven healthy adults (considered as an entire control group) were asked to walk while surface EMG signals were collected from five thigh muscles and three lower leg muscles on each leg (16 EMG channels in total). The 16-channel surface EMG data, recorded during a series of consecutive gait cycles, were simultaneously processed by multivariate empirical mode decomposition (MEMD), to generate fully aligned data scales for subsequent MMSE analysis. In order to conduct extensive examination of muscle coactivation complexity using the MEMD-enhanced MMSE, 14 data analysis schemes were designed by varying partial muscle combinations and time durations of data segments. Both TD children and healthy adults showed almost consistent MMSE curves over multiple scales for all the 14 schemes, without any significant difference (*p* > 0.09). However, distinct diversity in MMSE curve was observed in the CP group when compared with the control group. There appears to be diverse neuropathological processes in CP that may affect dynamical complexity of muscle coactivation and coordination during gait. The abnormal complexity patterns emerging in the CP group can be attributed to different factors such as motor control impairments, loss of muscle couplings, and spasticity or paralysis in individual muscles. This study expands our knowledge of neuropathology of CP from a novel point of view of muscle co-activation complexity, which might be useful to derive a quantitative index for assessing muscle activation characteristics as well as motor function in CP.

## Introduction

Cerebral palsy (CP) is a permanent disorder of movement and postural control that are non-progressive disturbances in the developing fetal or infant brain leading to primary and secondary lesions of the sensory, neuromuscular and musculoskeletal functions (Vaz et al., 2006; Rosenbaum et al., 2007; Franki et al., 2014). Clinical manifestations of these impairments are spasticity, dystonia, muscle contractures, bony deformities, incoordination, loss of selective motor control and weakness (Crenna, 1998; Gormley, 2001). However, the collective of these motor functional deficits cannot be effectively remedied by current medical treatment. Continuous exploration of their motor control mechanism alterations and development of clinical interventions to improve motor functions are always of great demand. In this regard, developing improved assessment strategies for quantifying abnormal manifestations as well as evaluating the effects of clinical intervention is the prerequisite (Lauer et al., 2007; Li et al., 2013).

There have been a great many methods aimed at evaluating motor function in children with CP as well as assessing the outcome of a clinical intervention. Among them, several classification scales are routinely used in clinic to measure motor function and functional impairment (Daltroy et al., 1998; Palisano et al., 2006). The use of these scales, however, is dependent on the skilled but subjective judgment of a clinician. Recent efforts have been made toward objective and quantitative examination of motor abnormalities and gait pathology in particular. For instance, a normalcy index (NI) has been put forward to distinguish differences in gait between subjects from the kinematic point of view (Romei et al., 2004). The gait deviation index (GDI), which has been described by recent studies (Schwartz and Rozumalski, 2008; Massaad et al., 2014), is a multivariate readily calculated method to quantify the pathological gait as a result of CP. Edinburgh visual gait score (EVGS) was presented by Read et al. (2003) as a three-dimensional gait analysis method based on computer vision; and its effectiveness and reliability in clinical diagnosis of abnormal gait were evaluated by Viehweger et al. (2010). Although these approaches and indices relying on kinematic data are able to provide a way to quantify motor deficits following neurological injuries, they are not directed toward characterizing the changes in muscle activation patterns. Alternatively, muscle biopsies, motor evoked potentials and intramuscular electromyographic (EMG) examinations can provide information about the muscle activation characteristics (Dietz et al., 1986; Rose et al., 1994). Their invasive feature, however, prevents the applications of these techniques especially on pediatric population.

Surface EMG is a non-invasive approach which measures muscle activity and thus can be used to evaluate the effect of rehabilitative treatment on muscle function (Drost et al., 2006). Previous studies reported routine use of surface EMG for clinical assessment and evaluation of motor impairment in CP (Gage, 1993; DeLuca et al., 1997). In most cases, the analysis of surface EMG data for motor function assessment has been done with relatively simple but useful parameters from the signal, such as signal amplitude, and variant in muscle onset and offset timing (Burridge et al., 2001; Tedroff et al., 2008; Frigo and Crenna, 2009; Bojanic et al., 2011). Recent studies have shown the potential of using multi-scale representations of surface EMG [derived from the wavelet analysis (Zhao et al., 2006; Istenic et al., 2010) or empirical mode decomposition (EMD) (Huang et al., 1998, 2003; Zhang et al., 2013)] other than single-scale parameters in the time or frequency domain, for better characterization of muscle activation patterns in both able-bodied adults and patients with neurological diseases. Specifically, Lauer et al. (2005, 2007) demonstrated the feasibility of applying continuous wavelet transform (CWT) techniques to surface EMG data for gait analysis and motor assessment in children with CP.

Recent advances in sensing technology allow for simultaneous recording of multiple surface EMG channels from a group of muscles (Drost et al., 2006). The usefulness of surface EMG recordings from multiple muscles has been demonstrated by various applications regarding muscle activity pattern identification during functional movements and gait in particular (Frigo and Crenna, 2009; Li et al., 2014). The limitation of these studies, however, has been that different EMG channels are individually processed in isolation, while the couplings across multiple muscles cannot be emphasized. On the contrary, simultaneously processing multiple EMG channels offers an opportunity to extract additional information regarding co-activation of multiple muscles. The capability of examining co-activation and coordination of muscles during a clinically relevant task could be potentially of great value.

Given the above, this study presents a novel application of the recently developed multivariate multi-scale entropy (MMSE) analysis on surface EMG signals to quantify muscle dysfunction in children with CP during gait, in terms of dynamical complexity of muscle co-activation. The applied MMSE technique is able to incorporate the multi-scale entropy (MSE) analysis within a multivariate data-adaptive framework (Ahmed et al., 2012). The MSE measures the basic dynamical complexity of a system over different time scales, which can be done by applying standard (single-scale and univariate) sample entropy (SampEn) analysis to intrinsic multiple data scales generated from input data by the EMD, a data-driven method (Hu and Liang, 2012). Recent multivariate extensions of both EMD and SampEn, namely multivariate EMD (MEMD) (Rehman and Mandic, 2010) and multivariate SampEn (MSampEn) (Ahmed and Mandic, 2011, 2012), have shown advanced performance in analysis of multi-channel physiological data, as compared with their standard univariate versions (Rehman et al., 2010; Ahmed et al., 2011; Mandic, 2011; Hu and Liang, 2012; Morabito et al., 2012). Taking both advantages, the technique used in this study, termed as MEMD-enhanced MMSE method by its proposer (Ahmed et al., 2012), operates in a fully multivariate manner, as it directly calculates MSampEn estimates for fully aligned scales generated by MEMD from multivariate input data. This technique applied to surface EMG data from multiple muscles ensures simultaneous analysis of their dynamical complexity, which offers analysis of muscle co-activation and coordination in a meaningful way. Therefore, the findings of the study can expand our knowledge of neuropathology of CP from a novel point of view of muscle co-activation complexity, which might serve as a potentially useful technique for assessment of motor function in CP.

## Materials and methods

### MMSE analysis method

#### EMD and MEMD

The EMD algorithm is a kind of fully data-driven and self-adaptive time-frequency domain analysis process which models the raw signal as a linear combination of a series of intrinsic oscillatory modes and a residual signal (Huang et al., 1998). The EMD decomposition of a time series $x{\left(t\right)}_{t\;=\;1}^{T}$ can be described as follow (Huang et al., 1998, 2003):

(1) $$x\left(t\right)=\sum_{i}^{N}c_{i}\left(t\right)+r\left(t\right)$$

where $c_{i}{\left(t\right)}_{t=1}^{T}$ represents the *i*-th intrinsic mode function (IMF), and $r{\left(t\right)}_{t=1}^{T}$ is the residual usually regarded to be the (*N* + 1)-th IMF. Consequently, the EMD method can be concisely described as follow:

(2) $$x\left(t\right)=\sum_{i}^{N\;+\;1}c_{i}\left(t\right)$$

These resultant IMFs are defined to present inherent oscillatory modes. However, when separately applied to multi-channel data, the standard EMD is likely to introduce mode-misalignment problem (Rehman and Mandic, 2010). In order to alleviate this problem, MEMD has been proposed recently to extend application of the standard EMD to multivariate data (Rehman and Mandic, 2010). With the benefit from the mode alignment property, the MEMD has been demonstrated to be suitable in analysis of non-stationary multivariate physiological time series (Rehman et al., 2010; Hu and Liang, 2012; Zhang et al., 2013).

The critical difference between MEMD and EMD is how to estimate the local mean. In EMD, local mean is an average of the upper and lower envelopes which can be obtained by interpolating the local maxima and the local minima of the signal. However, the parallel local maxima and local minima cannot be defined directly in the multivariate signal. Thus, in MEMD, multiple *n*-dimensional envelops are generated by taking signal projections along different directions in an (*n*-1)-dimensional space; then the local mean is obtained by calculating the average of these envelopes. Considering that ${\left\{\text{v}(t)\right\}}_{t\;=\;1}^{T}$ denotes *n*-variable time series, and $x^{\theta_{k}}$ is a set of vectors (indexed by *k*) along the directions represented by angles $\theta_{k}=\{\theta_{1}^{k},\theta_{2}^{k},\ldots ,\theta_{n-1}^{k}\}$ on an (*n*-1)-dimensional space, the MEMD algorithm is summarized as follows (Rehman and Mandic, 2010; Mandic, 2011):

1. Choose an appropriate point set based on Hammersley sequence for sampling on an (*n*-1)-dimensional space;
2. Compute a multidimensional projection $p^{\theta_{k}}\left(t\right){\}}_{t\;=\;1}^{T}$ of the multivariate input data ${\left\{\text{v}(t)\right\}}_{t\;=\;1}^{T}$ along a direction vector ${\text{x}}^{\theta_{k}}$, for all *k*, thus giving $p^{\theta_{k}}\left(t\right){\}}_{k\;=\;1}^{K}$ as the set of projections;
3. Locate the time points $t_{i}^{\theta_{k}}$ corresponding to the maxima of the set of projected signals $p^{\theta_{k}}\left(t\right){\}}_{k=1}^{K}$;
4. Interpolate $\left(t_{i}^{\theta_{k}},v(t_{i}^{\theta_{k}})\right)$, for each *k*, to get multivariate envelope curves $e^{\theta_{k}}{\left(t\right)}_{k\;=\;1}^{K}$;
5. For all *K* direction vectors, compute the mean m(*t*) of the envelope curves as follow:
  (3) $$m\left(t\right)=\frac{1}{K}\sum_{k\;=1}^{K}e^{\theta_{k}}(t);$$
6. Iterate on the detail **d**(*t*) = **v**(*t*)−**m**(*t*) until it becomes an IMF. Then the above procedure is applied to **v**(*t*)−**d**(*t*).

The stoppage criterion for multivariate IMFs is similar to that for the univariate IMFs presented in Huang et al. (1998). Considering that extrema cannot be properly defined for multivariate signals, the constraints for the number of extrema and zero crossings are not imposed (Rehman and Mandic, 2010). By projection, MEMD directly processes multivariate signals to produce the scale-aligned IMFs. The MEMD source code is publicly available from the webpage of it proposer (Rehman and Mandic, 2010)^1^.

#### SampEn and MSampEn

Entropy is an effective tool to measure the complexity and randomness of a dynamic system (Richman and Moorman, 2000; Costa et al., 2002, 2005). Among various entropy measures, sample entropy (SampEn) introduced by Richman and Moorman (2000) is an effective and robust one for the short and noisy time series. The SampEn, always used as a basic, single-scale and univariate entropy measure, has achieved successful applications in analysis of various physiological signals, such as diagnosis of cardiovascular diseases (Costa et al., 2002) and EMG activity detection (Zhang and Zhou, 2012).

In order to enable complexity analysis of multi-channel data, Ahmed and Mandic (2011) introduced MSampEn which takes into account both within- and cross-channel dependencies. For an *n*-variate time series ${\left\{x_{k,i}\right\}}_{i\;=\;1}^{N},\;k=1,\;2,\ldots ,n$, *k* = 1, 2,…, *n*, the calculation of MSampEn starts from the multivariate embedded reconstruction which is based on the composite delay vector:

(4) $$\begin{array}{l} X_{m}\left(i\right)=[x_{1,i},x_{1,i+\tau_{1}},\ldots ,x_{1,i+\left(m_{1}-1\right)\tau_{1}},x_{2,i}\\ x_{2,i+\tau_{2}},\ldots ,x_{2,i+\left(m_{2}-1\right)\tau_{2}},\ldots ,x_{n,i},\\ x_{n,i+\tau_{n}},\ldots ,x_{n,i+\left(m_{n}-1\right)\tau_{n}}], \end{array}$$

where $M=\left[m_{1},m_{2},\ldots ,m_{n}\right]\in R^{n}$ is the embedding vector, τ = [τ_1_,τ_2_,…,τ_*n*_] represents the time lag vector, the composite delay vector $X_{m}\left(i\right)\in R^{m}$ (where *m* = *m*_1_+*m*_2_+…+*m*_*n*_). Therefore, the MSampEn is calculated through the following procedures (Ahmed and Mandic, 2011, 2012):

1. Construct (*N*−δ) composite delay vectors $X_{m}\left(i\right)\in R^{m}$, where *i* = 1, 2, …,(*N*−δ) and δ = max{**M**} × max{**τ**}.
2. The maximum norm between any two composite delay vectors *X*_*m*_(*i*) and *X*_*m*_(*j*) is defined as the distance: *d*[*X*_*m*_(*i*), *X*_*m*_(*j*)] = *max*_*l* = 1,…, *m*_{|*x*(*i*+*l*−1)−*x*(*j*+*l*−1)|};
3. For a given *X*_*m*_(*i*) and a threshold *r*, count the number *P*_*i*_ of any pair of vectors that satisfies *d*[*X*_*m*_(*i*), *X*_*m*_(*j*)] ≤ *r*, *i*≠*j*, then calculate the frequency of occurrence $B_{i}^{m}\left(r\right)=P_{i}/\left(N-\delta -1\right)$, and define the average over all possible *i* ∈ [1, *N*−δ]:
  (5) $$B^{m}\left(r\right)=\frac{1}{N-\delta }\sum_{i=1}^{N-\delta }B_{i}^{m}\left(r\right).$$
4. Extend the dimensionality of multivariate delay vectors in Equation (4) from *m* to (*m*+1), then repeat the upper steps, and obtain the average *B*^*m*+1^(*r*) over all possible *i* ∈ [1, *n* × (*N*−δ)].

Finally, for a tolerance level *r*, the MSampEn is calculated as:

(6) $$MSampEn\left(M,\tau ,r,N\right)=-\text{ln}(\frac{B^{m\;+\;1}\left(r\right)}{B^{m}\left(r\right)}).$$

The MSampEn offers a quantitative approach for simultaneously analyzing the complexity of multi-channel data obtained from one physical process, which has been illustrated for chaotic physical phenomenon and physiological data (Ahmed and Mandic, 2011, 2012; Ahmed et al., 2011). The source code of MSampEn is also available from the webpage of its proposer (Ahmed and Mandic, 2012)^2^.

#### MEMD-enhanced MMSE

With MEMD acting as a multi-scale analysis tool to decompose input multivariate data into scale-aligned IMFs, the MMSE can be straightforwardly performed by applying MSampEn on those fully aligned scales (IMFs). Although the separate IMFs can directly represent multiple data scales, the “scales” for MMSE analyses in this study were defined to be cumulative IMFs: $c_{n}=\sum_{i\;=\;n}^{N}c_{i}$, where *n* ∈ [1, *N*] denotes the cumulative IMF index (or scale factor), and *c*_*i*_ denotes the *i*-th IMF of the multivariate data (Ahmed et al., 2012). Therefore, the MMSE analyses can be achieved by calculating and plotting the MSampEn measure given in (6), for each scale *n*, in a fine-to-coarse manner (Hu and Liang, 2012), which indicates that the multiple scales are produced in a way of consecutively removing the high-frequency (low-order) IMFs from the original input multivariate data (Ahmed et al., 2012).

### Subjects

Eleven children with CP (9 males, 2 females, age: 5.4 ± 2.2 years, mean ± standard deviation), eight children with typical development (TD) (3 males, 5 females, age: 6.6 ± 2.0 years) and seven healthy young adults (7 males, age: 24.7 ± 0.9 years) participated in the study. All 11 CP children were recruited from outpatient clinic of the neurological rehabilitation for children in the First Affiliated Hospital of Anhui Medical University (Hefei, Anhui Province, China), with approval from the association of ethics of the hospital. Inclusion criteria for children with CP participating in the study include: (1) age between 2.5 and 12 years old; (2) clinical diagnosis of CP; (3) ability to communicate with others; (4) experience of lower limb motor deficits that led to abnormal gait; (5) ability to walk independently or with assistance; (6) no history of other diseases that also lead to motor deficits; (7) no history of any kind of surgical therapies. The written consent was obtained from guardians of all CP children prior to the data collection experiments. Gross motor function classification system (GMFCS) (Palisano et al., 2006) was used by a physical therapist to assess motor function for each CP child. Demographic and clinical measures for the CP children are detailed in Table 1. All the 11 CP children were considered to form a CP group. In addition, 15 subjects, including eight age-matched TD children (also termed TD group) and seven healthy young adults (also termed AD group), were recruited through University of Science and Technology of China (Hefei, Anhui Province, China). The study was approved by the ethics committee of the university as well. All TD children were recruited from faculty families who understood and supported our study, while all AD subjects were graduate volunteers in the university. Both the TD and AD subjects were considered to form a healthy control group for examining abnormality in the CP group. All subjects in the control group had not suffered from any known neurological or orthopedic deficiencies. All TD children's legal guardians and AD subjects gave their informed consent before the experiment. The detailed information about the control group is listed in Table 2.

**Table 1:** **Clinical characteristics of subjects with CP, where F, female; M, male**.

**Subject**	**Gender**	**Age (years)**	**GMFCS**	**Diagnosis**
CP1	M	5.4	II	Spastic, diplegia
CP2	M	4.3	III	Spastic, diplegia
CP3	F	4.0	III	Spastic, diplegia
CP4	M	2.5	I	Spastic, right hemiplegia
CP5	M	7.0	I	Spastic, diplegia
CP6	M	8.6	II	Spastic, diplegia
CP7	F	9.8	I	Spastic, diplegia
CP8	M	4.8	II	Right hemiplegia
CP9	M	3.5	II	Right hemiplegia
CP10	M	5.5	II	Right hemiplegia
CP11	M	3.8	I	Right hemiplegia

**Table 2:** **Basic information of the control group, where F, female; M, male**.

**Subject**	**Gender**	**Age (years)**	**Subject**	**Gender**	**Age (years)**
TD1	F	7.6	AD1	M	23.2
TD2	F	9.2	AD2	M	25.7
TD3	M	9.5	AD3	M	25.4
TD4	M	6.0	AD4	M	24.8
TD5	M	6.0	AD5	M	24.3
TD6	F	5.2	AD6	M	24.2
TD7	F	4.5	AD7	M	25.5
TD8	F	4.5			

### Experimental protocol

**Figure 1:**
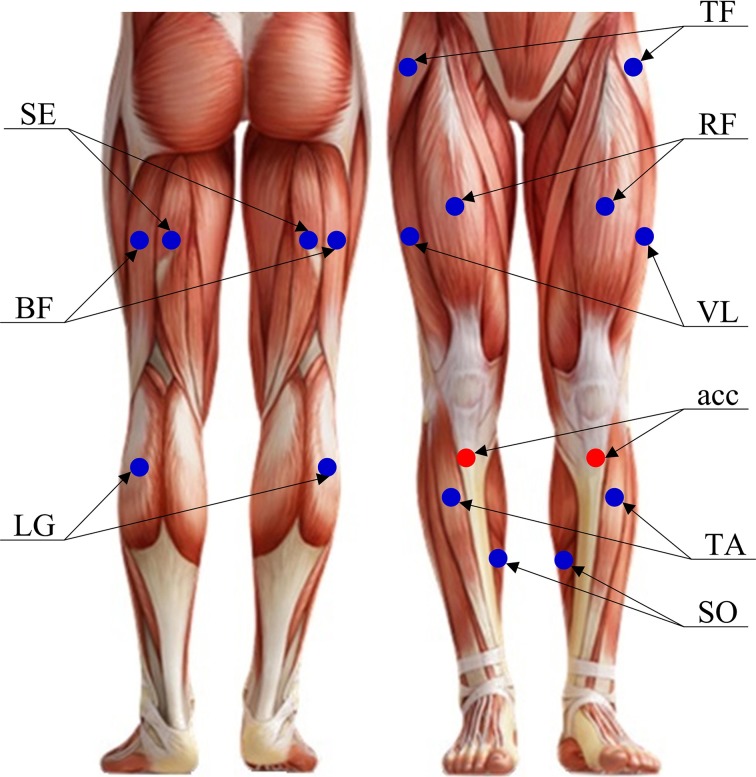
**The placement of 16 surface EMG sensors and two accelerometers over lower-extremity**.

**Figure 2:**
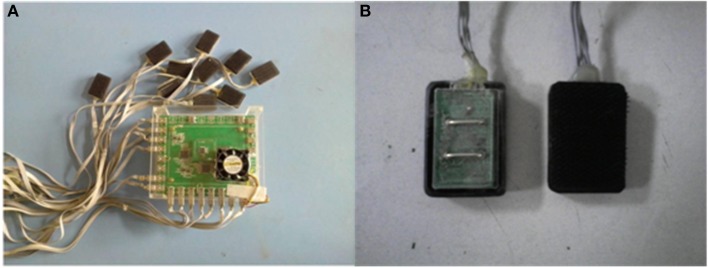
**A home-made portable multi-channel data recording system (A) and an individual surface EMG sensor (B)**.

Figure 1 shows placement of both surface EMG sensors and accelerometers used in this study. The surface EMG data were recorded bilaterally on the following muscles: (1) five thigh muscles including vastus lateralis (VL), rectus femoris (RF), semitendinosus (SE), biceps femoris (BF) and tensor fasciae latae (TF), and (2) three lower leg muscles including tibialis anterior (TA), soleus (SO) and lateral gastrocnemius (LG). These muscles were selected due to their relevance to gait. An individual surface EMG sensor was built with a pair of parallel bar-shape Ag-AgCl electrodes in a formation of 10 mm length, 1 mm width for each bar, and 10 mm spacing between bars to allow a bipolar channel of EMG recording, as shown in Figure 2. Each surface EMG sensor was placed in the middle of the targeted muscle belly, in a direction that ensured electrode bar perpendicular to the muscle fibers. After the skin surface was cleaned with a soft alcoholic pad, the EMG sensors were secured in place using both tape and stretchable wraps by an experienced experimenter. Besides, two tri-axis accelerometers were also bilaterally placed over the upper tibia below the knee (see Figure 1). For each accelerometer, only the axis along gravity was used to assist the determination of gait cycles.

For each trial of the experiment, the subjects were asked to walk at a self-selected speed across a straight 25-foot walkway. Two or three trials were necessary for each subject to ensure the recording of sufficient data. For some CP children who were not able to walk independently, their guardians were allowed to assist them in walking. By considering the safety of other CP children and TD children, they were followed by their guardians or experimenters. Sufficient rest was allowed between two consecutive trials for all subjects to avoid both muscular and mental fatigue, especially for the children with CP.

A home-made portable multi-channel data recording system (Figure 2A) was used to synchronously record surface EMG and acceleration (ACC) signals from two legs of subjects during gait. The sampling rate for each surface EMG channel was 1 kHz, while the acceleration signal was digitalized at 100 Hz. All data were recorded and stored to a laptop computer via USB for further analysis using a customized program in Matlab (ver. 2012, The Mathworks Inc., Natick MA, USA).

### Data analysis

#### Data preprocessing and segmentation

**Figure 3:**
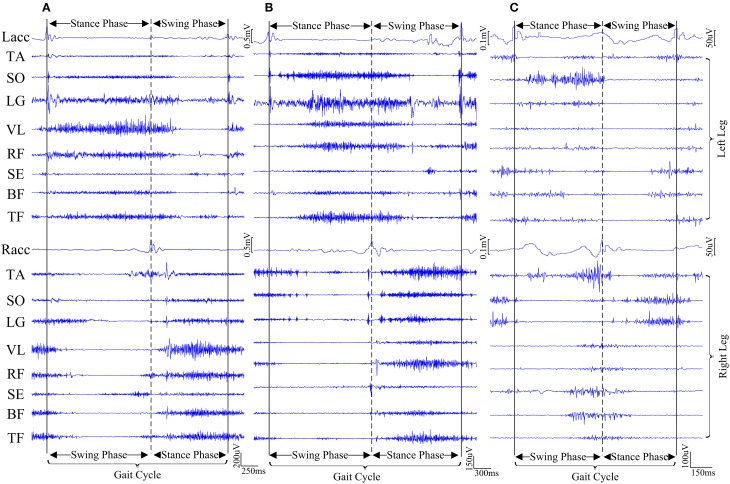
**Examples of representative surface EMG and acceleration signals approximately during one gait cycle from three subjects. (A)** CP1, **(B)** CP4, and **(C)** AD3. For each subject, the time duration between two vertical solid lines indicates one gait cycle, which is divided roughly into two gait phases by a vertical dashed line.

Both surface EMG and ACC signals recorded from lower-extremity were supposed to show cyclic patterns during gait. Figure 3 exhibits examples of raw data recorded from three subjects approximately during one gait cycle. It should be acknowledged that surface EMG signals recorded during walking showed clear cyclic pattern for most control subjects and a few CP children. However, for the majority of CP children, such cyclic pattern was not obvious due to their motor impairments and abnormal muscle activations, which caused difficulty in determining gait cycles for EMG signals. Therefore, the ACC signal along gravity was employed as additional reference, because the occurrence of each ACC peak indicates the moment that heel (or foot for some CP children) of the corresponding leg strikes the ground. Visual inspection was conducted on data across all trials to determine individual gait cycles (heel strike to heel strike) for all subjects. Furthermore, taking advantage of physiological characteristics during walking that both legs alternately make individual steps (two steps make up each gait cycle), each gait cycle can be roughly divided into a stance phase and a swing phase, given detected ACC peaks along the timeline from both legs. A stance phase of one leg occurs from an ipsilateral ACC peak to the next contralateral ACC peak, followed by a swing phase of the same leg corresponding to the remaining period of the same gait cycle. We also manually discarded any gait cycle contaminated by external interference like motion artifacts. For each subject, a series of gait cycles were determined, and corresponding EMG data segments were selected and concatenated along the timeline to form a 16-channel EMG data block.

#### MEMD analysis

The data blocks from all subjects were further concatenated as a 16-channel EMG dataset. This dataset and six channels of Gaussian white noise with the same length as the EMG dataset were reshaped into a 22-dimensional dataset which is suitable for MEMD analysis. Combining additional white noises with the original dataset is a technique to overcome the mode-mixing problem in MEMD analysis (Wu and Huang, 2009; Rehman and Mandic, 2010). A single MEMD operation was performed on the composite dataset, ensuring aligned scales across: gait cycles, channels/muscles; and different subjects.

**Figure 4:**
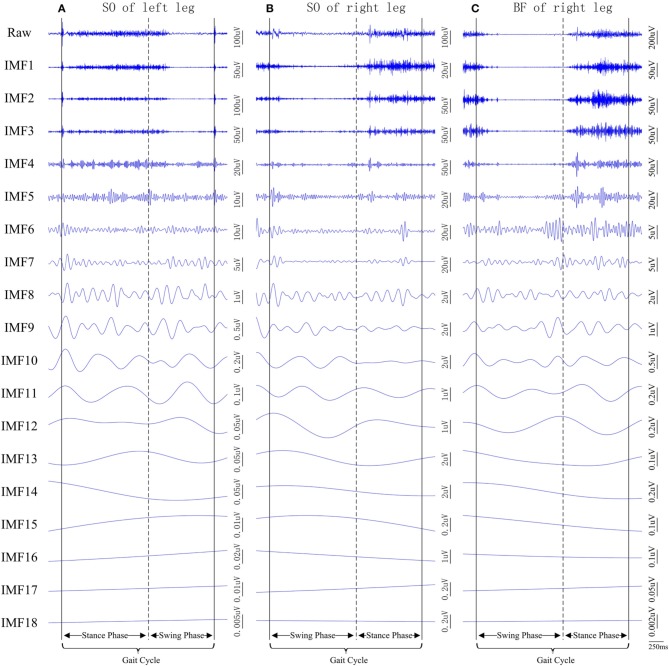
**Examples of raw surface EMG signal segments recorded from three muscles of the subject CP1 (also shown in Figure 3A). (A)** left SO, **(B)** right SO, and **(C)** right BF, and their corresponding IMFs after the MEMD.

In this study, MEMD analysis for the current 22-channel dataset (16 surface EMG channels combined with six white noise channels) produced 18 scale-aligned IMFs for each channel/muscle (see Figure 4). It was found that the first seven IMFs carried the majority of signal energy, whereas those with higher orders were considerably weaker. This was the case throughout the entire dataset. For this cause, the IMFs with order higher than six were summed up to represent a new single seventh order “IMF”. Note that the new seventh order IMF did not represent a proper IMF (according to its definition). The summation of all weaker IMFs at originally higher orders helped to simply data analysis at higher scales. Such summation process was equivalent to stopping the sifting process of MEMD implementation after first six iterations, while the resulting “residual” was regarded to be the seventh order “IMF.” This helped to save much computational power and did not change the scale alignment across channels. The resultant multivariate IMFs in a scale-aligned manner across multiple EMG channels facilitated the following examination of muscle co-activation complexity over multiple scales.

#### MEMD-enhanced MMSE analysis of muscle coactivation

**Figure 5:**
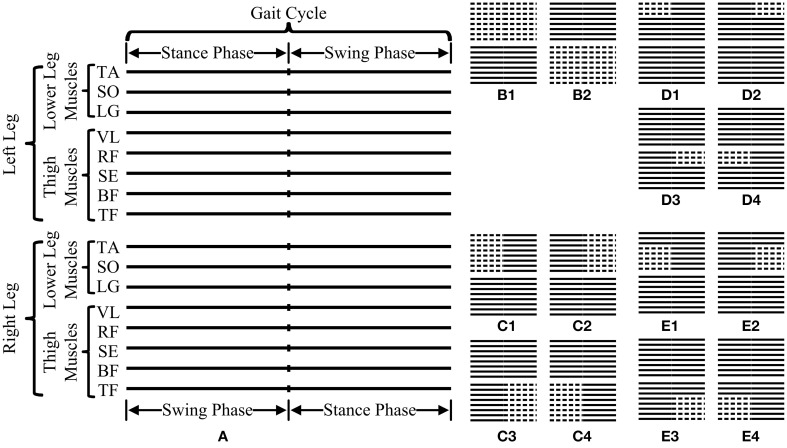
**Illustration of all 14 data analysis schemes used for MMSE analyses in this study. (A)** is a schematic diagram representing a basic data segment of surface EMG recordings from 16 muscles (represented by 16 parallel solid lines) over one gait cycle consisting of both labeled swing and stance phases. The 14 data analysis schemes can be categorized into four data organization strategies corresponding to **(B–E)**, respectively, where the dashed lines indicate the selected muscles and time segment for MMSE analyses.

Given the multiple scales adaptively generated by MEMD from the multivariate EMG data, MMSE analyses were subsequently performed by applying MSampEn to scale-aligned IMFs from a set of channels/muscles to reveal their coactivation complexity across multiple scales. Since the original EMG data were selected in a form of consecutive gait cycles, the MMSE analysis was performed on the data segment within each individual gait cycle to evaluate dynamic complexity of muscle coactivation during gait. Specifically, such evaluation approach was extensively conducted by varying partial muscle/channel combination and the time duration (an entire gait cycle or a gait phase). In this study, 14 data analysis schemes in total, derived from four data organization strategies, as shown in Figure 5, were proposed and briefly explained as follows:

1. The first strategy was used to group the EMG data from a single leg over an entire gait cycle, thus producing two data analysis schemes for both the left and right legs, as shown in Figures 5B1,B2, respectively.
2. The second strategy further considered the EMG data from a single leg over a certain gait phase other than an entire cycle. Therefore, four data analysis schemes were used when the EMG data from eight muscles of the left/right leg over a stance/swing phase were specifically selected for the MMSE analysis, as shown in Figures 5C1–C4, respectively.
3. In the third strategy, the EMG data from three lower leg muscles of one leg over a certain gait phase were selected, thus producing four data analysis schemes for the left/right leg and the stance/swing phase, as shown in Figures 5D1–D4, respectively.
4. In the fourth strategy, the couplings among five thigh muscles of one leg were further examined over a certain gait phase, thus similarly producing four data analysis schemes, as shown in Figures 5E1–E4.

Following each analysis scheme, a resultant MMSE curve (MSampEn estimates across different scales) was derived from the data within each gait cycle. Then, the average of these MMSE curves was calculated over all selected gait cycles as a representative MMSE curve for each subject. Consequently, the mean and SD of the MMSE curves, averaged over all subjects in a subject group, were computed as well to examine the difference of MMSE patterns between subject groups.

The parameters used for MSampEn calculation were set as *m*_*k*_ = 2, τ_*k*_ = 1 and *r* = 0.2 × *SD*, where SD represents the sum of standard deviations of the raw surface EMG time series all involved in the MSampEn calculation. The choice of these parameters followed recommendations from previous studies (Ahmed and Mandic, 2012; Ahmed et al., 2012) for good statistical reproducibility.

#### Statistical analysis

In order to identify changes in muscle coactivation complexity among subject groups and to examine the effect of scales, a series of separate Two-Way repeated-measure ANOVAs were applied on the MSampEn values for each of 14 data analysis schemes respectively, with the scale (7 levels) considered as the within-subjects factor and the subject group (3 levels) considered as the between-subjects factor. When necessary, *post-hoc* pairwise multiple comparisons with Bonferroni correction were used. The level of statistical significance was set to *p* < 0.05 for all analyses. All statistical analyses were carried out using SPSS software (version 16.0, SPSS Inc. Chicago, IL USA).

## Results

**Figure 6:**
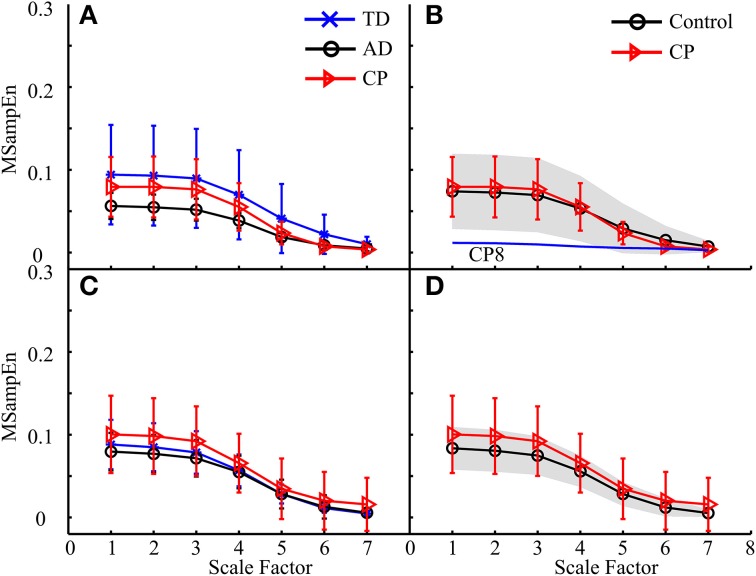
**The MMSE results for three subject groups (AD, TD, and CP) using the data analysis schemes shown in (A) Figure 5B1 and (C) Figure 5B2, respectively**. Each curve represents an average of MMSE curves over all subjects in the corresponding group, and error bars represent the SDs. When the TD and AD groups are combined to be one control group, the results were reproduced in **(B,D)**, respectively, where the gray area indicates the variation of MMSE results for the control subjects, with upper and lower boundaries equivalent to ± 1 SD deviation from the mean curve. In **(B)**, the MMSE curve of CP8 was specifically plotted due to its distinct deviation from those of other subjects (both control and other CP subjects).

The MMSE results for data from eight muscles of one leg during the entire gait cycle were shown in Figure 6. Figures 6A,C exhibit the MMSE results for three subject groups (AD, TD, and CP) using the data analysis schemes shown in Figures 5B1,B2, respectively. It can be observed that for both legs the MMSE curves from three subject groups simultaneously kept the same decreasing trend when the scale factor increased. For each of the left and the right leg shown in Figures 6A,C respectively, the ANOVA reported a significant effect of scale factor (*F* = 139.402, *p* < 0.001 for the left leg, and *F* = 84.486, *p* < 0.001 for the right leg) on MSampEn values. However, neither significant effect of the subject group (*F* = 0.720, *p* = 0.498 for the left leg, and *F* = 1.860, *p* = 0.178 for the right leg) nor interaction between two factors (*F* = 0.509, *p* = 0.906 for the left leg, and *F* = 1.616, *p* = 0.094 for the right leg) was observed for any of two legs.

**Figure 7:**
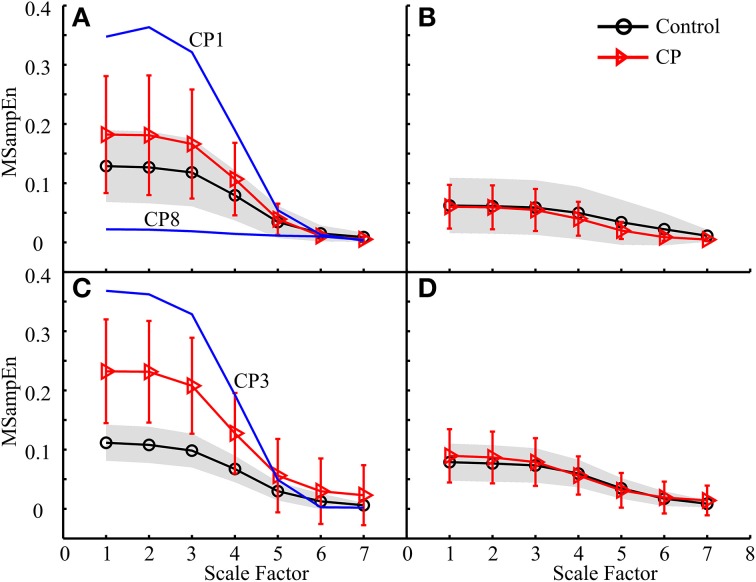
**MMSE results for both the control and CP subject groups using the data analysis schemes shown in (A) Figure 5C1, (B) Figure 5C2, (C) Figure 5C3, and (D) Figure 5C4, respectively**.

**Figure 8:**
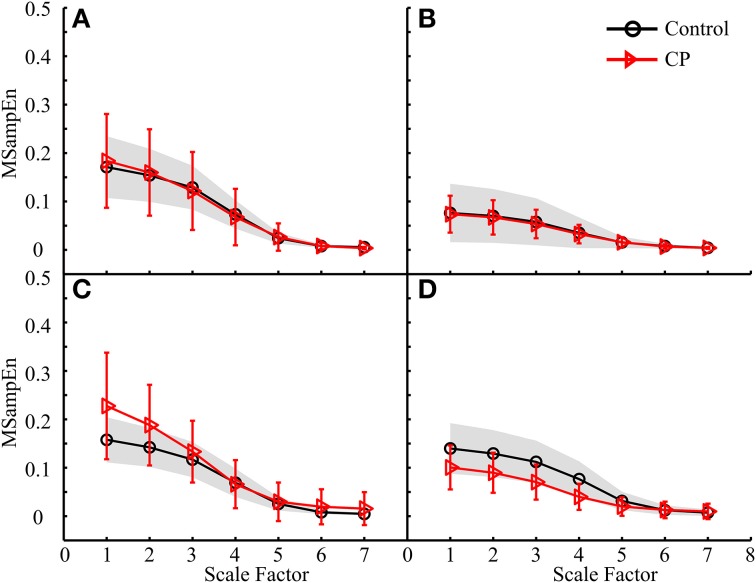
**MMSE results for both the control and CP subject groups using the data analysis schemes shown in (A) Figure 5D1, (B) Figure 5D2, (C) Figure 5D3, and (D) Figure 5D4, respectively**.

**Figure 9:**
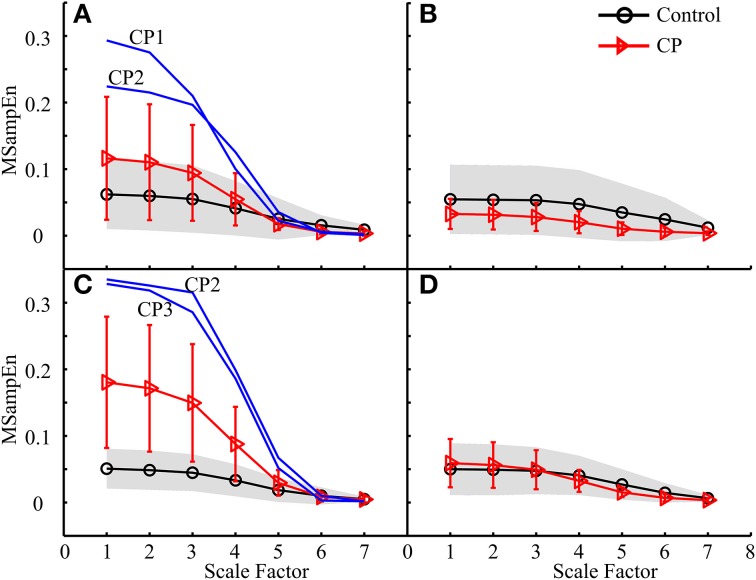
**MMSE results for both the control and CP subject groups using the data analysis schemes shown in (A) Figure 5E1, (B) Figure 5E2, (C) Figure 5E3, and (D) Figure 5E4, respectively**.

Specifically, no significant difference between the TD and AD groups (*p* > 0.09) was always the case for the following analyses. For this cause, the TD and AD groups were combined as an integrated control group in order to simplify following analyses. We also reproduced the MMSE results by (1) using a gray shading area to indicate the MMSE variation (± 1 *SD*) for the control group, and (2) individually plotting the MMSE curves of specific CP children which distinctly deviated from the mean curves of both control and CP groups, if applicable. This facilitated our examination of possible differences in MMSE results between CP and control groups and abnormalities of individual CP children. The MMSE results shown in Figures 6A,C, for example, were reproduced in Figures 6B,D. Following the same way of exhibiting MMSE results, the results derived from the other three data organization strategies (the second, third and fourth) are shown in Figures 7–9, respectively. All these experimental results can be summarized as follows:

1. For all analyses, the mean MMSE curves for both control and CP groups showed the same decreasing trend, almost approaching to zero at the scale factor 7. The ANOVA revealed a significant effect of the scale factor on the MMSE results (*p* < 0.001). More specifically, the MSampEn values decreased rapidly at the scale factors from 3 to 5, for most MMSE curves.
2. The MMSE results derived from some data analysis schemes displayed different mean curves for the control and CP groups, as shown in Figures 7C, 8D, 9C, with statistical significance at scale factors lower than 5 (*p* < 0.039). More specifically, it can be found that the CP group had significantly higher MSampEn values over the first four scales in the Figure 7C (*p* = 0.003) and Figure 9C (*p* < 0.001), and significantly lower MSampEn values over the first four scales in the Figure 8D (*p* = 0.039), than the control group.
3. From MMSE results derived from some other data analysis schemes, the CP group exhibited obviously larger variation (SD) in MMSE results than the control group, although no significant difference can be found between mean MMSE curves of both groups, as shown in Figures 6D, 7A,D, 8A,C, 9A.
4. Relatively abnormal patterns of MMSE curve, which deviated from mean curves of both control and CP groups, appeared in individual CP subjects (i.e., CP1-3 and CP8), when some specific data analysis schemes were applied. All these curves were specifically shown in Figures 6–9.

## Discussion

Applying an entropy measure to multiple scales derived from input data by the coarse-grained approach (Costa et al., 2002, 2005; Ahmed and Mandic, 2011, 2012; Morabito et al., 2012), wavelet analysis (Rosso et al., 2001; Zhao et al., 2006; Istenic et al., 2010) or EMD method (Hu and Liang, 2012; Zhang et al., 2013), is the key feature of the multi-scale entropy analysis, which has been successfully applied to identification of different real-world biomedical time series in terms of their dynamical complexity (Costa et al., 2002, 2005; Zhang et al., 2013). The EMD is a recently developed method that is able to generate multiple data scales (namely IMFs), to be used for the subsequent MSE analysis (also termed EMD-based MSE, or IMEn) with improved performance owing to fully data-driven nature of the EMD (Hu and Liang, 2012; Zhang et al., 2013). As its generalized multivariate extension, MEMD directly operates on multivariate signals to produce the same number of mode-aligned scales (i.e., multivariate IMFs) across multiple data channels, thus facilitating the analysis of their properties at the same scale. With the advanced MEMD, however, the MSE methods given in Hu and Liang (2012) and Zhang et al. (2013) still employed univariate SampEn to process individual channels. By contrast, MSampEn was a recently introduced multivariate extension of SampEn, which enables complexity estimates for multi-channel data by additionally taking into account couplings across multiple channels (Ahmed et al., 2011; Ahmed and Mandic, 2011, 2012; Morabito et al., 2012; Wei et al., 2012). Given both MEMD and MSampEn, a fully multivariate framework for assessing dynamical complexity of multi-channel data over different scales, termed MEMD-enhanced MMSE analysis, was consequently proposed by Ahmed et al. (2012). Taking advantage of its fully multivariate property, a novel application of the MEMD-enhanced MMSE method to multivariate surface EMG recordings from multiple muscles allows meaningful analysis of muscle co-activation and coordination in term of non-linear dynamic complexity. This study presents such application for multiple lower-limb muscles during gait, with the purpose of providing new insight into the mechanism of motor control system during walking as well as characterizing abnormal gait in children with CP.

The decreasing trend of MMSE results found for any subject group and any applied data analysis scheme indicated that the muscle co-activation complexity decreased when successively removing the low-order IMFs (high-frequency components) from the original surface EMG input. At the scale factors higher than 5, the MSampEn value was likely to fall down to a low level approximating to zero, demonstrating that the primary signal components were distributed over first five IMFs after the MEMD decomposition. This finding also confirmed necessity of only obtaining the first six IMFs (scales) via MEMD implementation.

Many previous studies employed muscle synergy analysis (Artoni et al., 2013; Li et al., 2013) and postural control analysis (Mercer and Sahrmann, 1999; Sundermier et al., 2001), reporting differences in gait pattern between typically developed children and healthy adults. Such differences were probably attributed to the development of gait maturity, that is, the gait patterns for healthy adults are considered as being mature, whereas immature gait patterns exist in children with typical development. In this study, however, both TD and AD groups yielded consistent MMSE curves across all designed data analysis schemes without significant difference at any scale. Our finding truly accords with the general understanding of “normal” gait in healthy controls regardless of age. This may be explained by the reason that the MEMD-enhanced MMSE method, at least the applied MMSE approach including all 14 data analysis schemes, is very likely to be insensitive to the development of gait maturity. On this basis, it is straightforward to use the MMSE results derived from the integrated control group as normal reference that helps to assess abnormality in CP.

Following the data analysis schemes in Figures 5C3,E3, the MMSE results in Figures 7C, 9C revealed that the CP group had increased MSampEn at the first four scales with statistical significance (*p* < 0.003), as compared with the control group. Besides, it can also be found that Figures 7A, 9A, corresponding to data analysis schemes in Figures 5C1,E1, exhibited relatively higher MSampEn values at the first four scales for the CP group than the control group, despite of no statistical significance (*p* = 0.320 in Figure 7A and *p* = 0.209 in Figure 9A). In these cases, especially thigh muscles of both legs were involved to examine their activity over the time of stance phase of a gait cycle. The increased complexity of these muscles reflects abnormally high correlation among them, which can be attributed into motor control impairments in the CP group. This might also be a direct reflection of substantial over-activation, abnormal synchronization and spasticity in the examined combination of muscles, as a result of brain injury following CP. It has been reported from the literature (Crenna, 1998; Lauer et al., 2007; Bar-On et al., 2014) that increased recruitment of active motor units and sustained motor unit firing exist in over-activation of spastic muscles. In this regard, the MMSE results reported in Figures 7C, 9C revealed that the prevalence of thigh muscle spasticity in the CP children dominantly affected their gait patterns during the stance phase of the ipsilateral leg, and that such impairment was much severer on the right side than the left side by comparing results in Figure 7C with Figure 7A and Figure 9C with Figure 9A. This was agreed with the clinical diagnoses of right hemiparesis for almost half of the CP group, while others were children with spastic diplegia.

By contrast, the MMSE results in Figure 8D reported that the CP group had significantly lower MSampEn values at the first four scales than the control group (*p* = 0.039). Its corresponding data analysis scheme shown in Figure 5D4 examined the activities of three lower leg muscles of the right leg during the swing phase of the ipsilateral leg, which was assumed to contribute to posture stabilization and regulation other than direct gait formation. The reduced muscle coactivation complexity in CP children can be explained by two possible reasons. One is function deficits in individual muscles as a result of muscle paralysis with insufficient motor output (Gormley, 2001). The other is loss of muscle couplings. Specifically, this finding shows a loss of dynamical neuromuscular responsiveness to the external and environmental conditions (e.g., interference to posture balance), which supports the more general concept of multi-scale complexity loss with disease (Costa et al., 2002, 2005).

Under some data analysis schemes, although no significant difference was found when comparing the mean MMSE curves from both the control and CP groups, much larger variation emerged in the complexity measures over the first four scales for the CP group than the control group (see Figures 6D, 7A,D, 8A,C, 9A). Due to the factors discussed above, both increased and decreased complexity might be found among individual CP children, which led to large variation of their resultant MMSE curves. This finding also demonstrates diversity of motor impairments following CP (Rosenbaum et al., 2007).

Besides the pooled data analysis of gait abnormality in CP population, the possibility of assessing the gait pattern based on MMSE results for individual CP subjects can be demonstrated as well. The factors that resulted in abnormal MMSE patterns in the CP population can also be used to explain the MMSE results for specific subject with CP. For example, three CP children, CP1–CP3, had extraordinarily increased complexity in Figures 7A,C, 9A,C. This can be explained by over-activation of the leg muscles induced by the gait, as a result of prevalence of muscle spasticity. The MMSE results for these three subjects were consistent with their clinical diagnosis of spastic diplegia with relatively high levels of GMFCS (level III for the CP2 and CP3, level II for the CP1), demonstrating the severest motor impairments among all of the tested CP children. Another example is associated with the CP8, who presented a representatively low and flat MMSE curve over all scales in Figures 6B, 7A, indicating reduced couplings and coordination of the muscles on the left side, especially during the stance phase. This was agreed with the clinical observation of leg muscle paralysis for the subject CP8.

By comparing the MMSE results between 14 used data analysis schemes, it can be found that more distinct difference between subject groups was likely to emerge when the analyses were performed within a specific gait phase (see Figures 7–9) than the entire gait cycle (see Figure 6). These findings accord with the biomechanical principle that different muscles periodically activate during specific phases in a gait cycle to coordinate gait (Frigo and Crenna, 2009). Therefore, examination of muscle coactivation according to gait phases is a practical way to discern specific gait abnormalities. In this regard, further dividing a gait cycle into four phases, namely stance, heel-off, swing, and heel-strike (Pappas et al., 2001), would help the quantitative analysis of gait in CP, which constitutes our future work.

Also, it should be noteworthy that the application of MMSE method in this study was just a global analysis of a group of muscles in terms of their co-activation complexity over multiple scales. We acknowledge that there are an enormous number of possible combinations of muscles/channels for the MMSE analyses, where the 14 data analysis schemes were selected for a preliminary and basic investigation. This remains a limitation for the current study. Moreover, pooled data analysis of CP children and healthy control subjects was performed to reveal the difference in muscle co-activation complexity between the CP and control groups during walking. The significance at an individual subject level, however, was not demonstrated. The possible evolution of the MMSE method used in this study toward clinical diagnosis or impairment assessment requires a larger study with many more children with CP to quantitatively assess differences between individual subjects.

In conclusion, this study presents dynamical complexity analysis of muscle coactivation during gait by applying a recently developed MEMD-enhanced MMSE method on surface EMG signals bilaterally acquired from 16 lower extremity muscles. The MMSE results were consistent between TD children and healthy adults in control group. On the contrary, various patterns of MMSE curve, which were different from those in the control group, can be observed in CP group. There appears to be diverse neuropathological processes in CP that may affect the complexity of muscle coactivation and coordination during gait. The abnormal complexity patterns emerging in CP group can be attributed to different factors such as motor control impairments, loss of muscle couplings, and spasticity or paralysis in individual muscles. The methodology presented in this study provides a supplementary way to quantitatively assess the motor impairments of CP; and it helps to better understand motor control mechanism alterations as well as neuropathology of the disease.

## Conflict of interest statement

The authors declare that the research was conducted in the absence of any commercial or financial relationships that could be construed as a potential conflict of interest.

## Footnotes

^1^Rehman, N., and Mandic, D. P. (2010). (Online). Available online at: http://www.commsp.ee.ic.ac.uk/~mandic/research/emd.htm

^2^Ahmed, M. U., and Mandic, D. P. (2012). (Online). Available online at: http://www.commsp.ee.ic.ac.uk/~mandic/research/Complexity_Stuff.htm
